# Effects of various extraocular muscle enlargement patterns on muscle diameter index in graves ophthalmopathy patients: a retrospective cohort study

**DOI:** 10.1038/s41598-023-43942-w

**Published:** 2023-10-07

**Authors:** Junjie Yang, Jin Chen, Bingjie Shi, Yayan You, Xiaohuan Pi, Guang Zhao, Fagang Jiang

**Affiliations:** 1 Department of Ophthalmology, Union Hospital, Tong Medical College, Huazhong University of Science and Technology, Wuhan, China; 2 Wuhan Sixth Hospital, Wuhan, China; 3 Solution (Shanghai) Information Technology Co., Shanghai, China

**Keywords:** Diseases, Risk factors, Signs and symptoms

## Abstract

Graves ophthalmopathy (GO) patients often undergo retrobulbar injection of glucocorticoids (GCs) as a common therapeutic approach. This study aimed to explore the impact of various patterns of extraocular muscle (EOM) enlargement on EOM changes following retrobulbar GCs injection in patients with GO. A retrospective analysis was conducted on GO patients who underwent retrobulbar GCs injections. Data pertaining to EOM diameter (EMD) and muscle diameter index (MDI) were collected from orbital computed tomography (CT) scans. The MDI change (ΔMDI) was calculated by comparing pre- and post-injection MDI values. The relationship between each pre EMD/MDI and ΔMDI was assessed using univariate and multivariate logistic regression analysis. A total of 68 patients with GO were included in this study, accounting for 118 eyes. After retrobulbar injections of GCs, 84 eyes showed a decrease in the MDI, while 34 eyes exhibited an increase in MDI. A threshold effect was observed in the relationship between medial pre EMD/MDI and ΔMDI. When the medial pre EMD/MDI was less than 0.28, a higher medial pre EMD/MDI was associated with a smaller ΔMDI (β = − 25.21, *p* = 0.0175). However, when the medial pre EMD/MDI was greater than 0.28, no significant association was found between pre EMD/MDI and ΔMDI. There was a negative correlation between medial + lateral pre EMD/MDI and ΔMDI (β = − 11.76, *p* < 0.0189). A higher medial + lateral pre EMD/MDI was associated with a greater decrease in MDI. Additionally, there was a positive correlation between superior rectus muscle-levator complex (SRLC) pre EMD/MDI and ΔMDI (β = 11.92, *p* = 0.040). The higher the value of SRLC pre EMD/MDI, the greater the ΔMDI. There was an association between pre EMD/MDI and changes in EOMs after retrobulbar injection of GCs in GO patients. In patients with predominantly enlarged medial rectus muscles and severe degrees of enlargement, retrobulbar injection of GCs should be assessed for its benefit; a combination of medial and lateral rectus muscle enlargement is beneficial for the shrinkage of EOMs following retrobulbar injections; the involvement of the SRLC rectus muscle may be a disadvantageous pattern of shrinkage of EOMs following retrobulbar injections.

*Trial registration* This study is retrospectively registered. We have registered this study with the Chinese Clinical Trials Registry (www.chictr.org.cn, registration number: ChiCTR2200063429).

## Introduction

Graves’ ophthalmopathy (GO) is a chronic autoimmune disease characterized by the enlargement of extraocular muscles (EOMs)^1–3^. Computed tomography (CT) has proven to be a valuable tool for evaluating EOMs^4,5^. Different patterns of extraocular muscle enlargement and fatty hyperplasia were observed in patients with GO^6^.

A major therapeutic agent for GO is glucocorticoids (GCs). A number of studies have been conducted on the use of retrobulbar injections of GCs due to their low systemic side effects^7–10^. Ebner et al. reported in a prospective, multicenter, single-blind, placebo-controlled randomized controlled trial that retrobulbar administration of trimethoprim (TA) reduced diplopia symptoms and EOMs volume in patients with GO without adverse systemic or ocular effects^7^. Wang et al. conducted retrobulbar injection of GCs in 386 patients with GO (515 eyes) and assessed the outcome. They found that 30.21% of the patients showed improvement in their ophthalmopathy^10^. In our clinical experience, we have also observed varying responses in EOM volume reduction following retrobulbar injections of GCs. This variability has drawn our attention.

The muscle diameter index (MDI) refers to the sum of the diameters of the SRLC, inferior rectus, medial rectus, and lateral rectus muscles^11^. According to Zsolt et al. extraocular muscle diameter (EMD) is considered a reliable measure of muscle volume^12,13^. In this study, we aimed to differentiate different patterns of EOM enlargement in GO patients using the EMD/MDI ratio. The purpose of this study is to determine the effect of different EOM enlargement patterns on changes in EOMs following retrobulbar injection of GCs.

We conducted CT measurements of GO patients before and after retrobulbar injection to obtain the EMD and MDI values. By comparing the two MDI values, we calculated the ΔMDI, representing the change in EOMs after retrobulbar injection. Through analyzing the correlation between pre EMD/MDI and ΔMDI, we hoped to gain insights into the impact of distinct EOM enlargement patterns on EOM alterations following retrobulbar injection of GCs.

Our study aimed to provide valuable information to guide clinical decision-making, offering more targeted and effective treatment strategies for individual GO patients.

## Materials and methods

### Study design

In this retrospective cohort study, we conducted data collection of patients admitted to the ophthalmology department at Union Hospital, Tongji Medical College, Huazhong University of Science and Technology from November 1, 2016, to November 1, 2022. A total of 146 patients were initially included in this study, and eventually, a total of 68 cases (118 eyes) were enrolled in the final analysis. These patients received retrobulbar injections of glucocorticoids. The retrobulbar injection of GCs contained 20 mg of triamcinolone acetonide (TA) and 2.5 mg of dexamethasone.

The inclusion criteria we used included the following. (a) Age between 18 and 80 years; (b) The patient was treated with retrobulbar GCs injection during his or her inpatient stay in our ophthalmology department; (c) Orbital CT within 1 week prior to retrobulbar GCs injection, and repeat orbital CT within 2 months after the final injection; (d) Patient received low-dose (0.5 mg/kg) oral maintenance treatment with methylprednisolone tablets after injection, except for not receiving other treatment; (e) Patients were diagnosed with GO and had a Clinical Activity Score (CAS) of ≥ 3, with one or more rectus muscle enlargements visible on CT scan. (f) Prior to injection, patients had intraocular pressure (IOP) between 10 and 21 mmHg in both eyes, and IOP did not exceed 21 mmHg after injection.

The exclusion criteria are as follows. (a) Age < 18 years or age > 80 years; (b) History of ocular trauma or surgery; (c) Use of GCs or radiation therapy within the past 6 months; (d) Women who are pregnant; (e) The lack of data for the general examination and the CT scan. (f) CAS < 3, or no rectus muscle enlargement observed on CT scan. (g) IOP higher than 21 mmHg before or after each injection.

### Data collection

Patient information and CT images of the included patients were collected using the electronic medical record system of the Union Hospital, Tongji Medical College, Huazhong University of Science and Technology. The CT data was obtained using an image archiving and communication system, which is a networked system for storing images. Three separate measurements were made of each index, and the average of the three measurements was calculated. Additionally, data were collected and managed using the EDC data acquisition and management system (study.empoweredc.com, Shanghai, China).

### Retrobulbar injection procedure

The medication dose used in this study was a combination of 20 mg of TA and 2.5 mg of dexamethasone, administered through retrobulbar injection. The injection method involved placing the patients in a supine position and cleansing the eyelids with povidone-iodine solution. The mixture of corticosteroids was slowly injected by the same physician using a disposable syringe (22–27 gauge, 3 cm long), with the needle inserted vertically into the lower outer quadrant of the orbit. After penetrating approximately 1.5 cm, and then directed medially and cephalad toward the apex of the orbit inserted an additional 1.5 cm. avoid the eyeball and surrounding blood vessels. Following a negative aspiration for blood, slowly inject of medication, and the needle was left in place for 10 s before withdrawal. The injection site was disinfected, covered with gauze, and pressed for one minute to prevent hematoma formation. After one minute, the gauze was removed, and the patient was asked to open their eyes for examination of visual acuity and orbit pressure. After confirming no abnormalities, the injection site was disinfected again, covered with gauze, and then bandaged. Return to the ward and inject 250 ml Mannitol intravenously once. IOP measurements were taken before the injection and on the next day after the injection. A baseline orbital CT scan was performed before the first injection, followed by injections at one-month intervals for a total of three injections. After completing three injections, a follow-up orbital CT scan was performed.

### Extraocular muscle diameter (EMD) measurements

CT data was acquired using a Picture Archiving and Communication System (PACS), and the window level/window width was set at 60/300 Hounsfield units (HU) to optimize visualization of the EOM. Each EMD measurement was performed three times by the same researcher, and the average value was taken to ensure accuracy and reduce measurement variability.

- EMD measurements: axial CT measurements showed the horizontal diameter of the medial rectus and lateral rectus muscle at the widest part of the muscle belly.
- Sagittal CT measurements showed the vertical diameter of the superior rectus muscle-levator complex (SRLC) and inferior rectus muscle at the widest part of the muscle belly, where the superior rectus and levator muscles were considered as one muscle group and measured together.
- MDI was defined as the sum of EMD of SRLC, inferior rectus, medial rectus, and lateral rectus muscles.
- ΔMDI was defined as the post-injection MDI minus the pre-injection MDI.

Patients whose MDI decreased (ΔMDI < 0) after retrobulbar GC injection were categorized into the shrinkage group. Patients whose MDI remained unchanged or increased(ΔMDI ≥ 0) after retrobulbar GC injection were categorized into the non-shrinkage group.

### Statistical analysis

Statistical analysis was performed with R version 4.0.3 (https://www.R-project.org) and EasyR (https://www.easyr.cc Solutions, Inc., Shanghai). A two-tailed *p*-value of < 0.05 was considered statistically significant in all analyses. Quantitative data were described by the mean ± standard deviation. Statistical descriptions of qualitative data were described using frequencies (%).

To assess the normality of the measured data, the Kolmogorov–Smirnov normality test was performed. For data that followed a normal distribution, the t-test was used to compare the two groups; otherwise, the Wilcoxon rank sum test was applied. Categorical data were analyzed using the chi-square test to determine the significance of differences between groups.

In order to explore the relationship between specific EMD/MDI and ΔMDI, a logistic regression analysis was conducted. Additionally, we considered the possibility of a nonlinear relationship between pre EMD/MDI and ΔMDI, and therefore, we used a generalized additive model to identify any potential nonlinearities. If a nonlinear relationship was detected, a dichotomous linear regression model was used to estimate the threshold between them.

### Ethics approval and consent to participate

This study was approved by the Human Ethics Committee of Union Hospital, Tongji Medical College, Huazhong University of Science and Technology. It was carried out according to the Declaration of Helsinki. We have registered this study with the Chinese Clinical Trials Registry (www.chictr.org.cn, registration number: ChiCTR2200063429). Due to the retrospective nature of the study and the de-tracking and anonymization of various patient data by the cohort, informed consent was waived by the Human Ethics Committee of Union Hospital, Tongji Medical College, Huazhong University of Science and Technology.

## Results

### Patients’ characteristics

A total of 146 patients were initially included in this study. There are 6 adolescents among them, 12 patients with a history of trauma to their eyes and ocular surgery in the previous six months, 29 patients with GCs and radiotherapy within six months, 20 patients with incomplete CT data and 11 patients with IOP increased greater than 21 mmHg after injection. At the conclusion of the final analysis, there were 68 patients with 118 eyes.

The study included 34 men (50%) and 34 women (50%). The mean age of the included patients was 50.86 ± 9.67 years. Among them, 35 patients had hyperthyroidism (56%), 1 patient had hypothyroidism (3%), 5 patients had thyroid tumors (8%), and 21 patients had no history of thyroid-related diseases (34%). Out of the 118 eyes, 50 had bilateral involvement, and 18 had unilateral involvement. Following retrobulbar injection of GCs, the MDI decreased in 84 eyes and increased in 34 eyes (Table 1).

**Table 1:** Basic Characteristics of Study Participants.

	Pre-injeciton	Post-injection	*P*-value*
Men(%)	34 (50%)		
Age(year)	50.86 ± 9.67		
SRLC EMD(mm)	5.72 ± 1.94	5.35 ± 1.79	0.131
Inferior EMD(mm)	6.55 ± 1.88	5.94 ± 1.69	0.009
Medial EMD(mm)	6.06 ± 2.36	5.71 ± 2.25	0.246
Lateral EMD(mm)	4.34 ± 1.81	4.19 ± 1.63	0.505
SRLC EMD/MDI	0.25 ± 0.06	0.28 ± 0.06	< 0.001
Inferior EMD/MDI	0.29 ± 0.06	0.25 ± 0.05	< 0.001
Medial EMD/MDI	0.26 ± 0.06	0.27 ± 0.06	0.203
Lateral EMD/MDI	0.19 ± 0.05	0.20 ± 0.05	0.127
MDI(mm)	22.6 ± 6.01	21.18 ± 5.56	0.062
ΔMDI#		− 1.49 ± 3.77	
ΔMDI grouping			
< 0 (MDI decreased)		84 (71.19%)	
≥ 0 (MDI increased)		34 (28.81%)	

Values are expressed as means ± standard deviation, or numbers (percentages). *Paired t-test; #ΔMDI was defined as the post-injection MDI minus the pre-injection MDI.

### Univariate logistic regression analysis of Pre EMD, Pre EMD/MDI, and ΔMDI

Univariate logistic regression analysis was conducted for pre EMD, pre EMD/MDI, and ΔMDI (Table 2). Our analysis revealed that each pre EMD was negatively correlated with the ΔMDI: SRLC pre EMD (β = − 0.38, *P* = 0.0345), inferior pre EMD (β = − 0.60, *p* = 0.0010), medial pre EMD (β = − 0.57, *p* < 0.0001), and lateral pre EMD (β = − 0.57, *p* < 0.0001). This indicates that higher respective pre EMD before injection were associated with a greater decrease in MDI.

**Table 2:** Univariate Logistic Regression Analysis of Pre EMD, Pre EMD/MDI, and ΔMDI.

	β	95% CI	*P*-value
SRLC pre EMD(mm)	− 0.38	(− 0.73, − 0.03)	< 0.0001*
Inferior pre EMD(mm)	− 0.6	(− 0.95, − 0.25)	0.001*
Medial pre EMD(mm)	− 0.57	(− 0.84, − 0.30)	< 0.0001*
Lateral pre EMD(mm)	− 0.92	(− 1.27, − 0.58)	< 0.0001*
SRLC pre EMD/MDI	11.92	(0.67, 23.18)	0.0401*
Inferior pre EMD/MDI	3.49	(− 7.43, 14.41)	0.5321
Medial pre EMD/MDI	− 5.75	(− 17.27, 5.76)	0.3295
Lateral pre EMD/MDI	− 16.08	(− 30.25, − 1.92)	0.028*
Medial + lateral pre EMD/MDI	− 11.76	(− 21.43, − 2.09)	0.0188*

*P < 0.05.

Furthermore, we observed a positive correlation between SRLC pre EMD/MDI and ΔMDI (β = 11.92, *p* = 0.0401), indicating that greater SRLC pre EMD/MDI were associated with larger ΔMDI. In contrast, there was a negative correlation between lateral pre EMD/MDI and ΔMDI (β = − 16.08, *p* = 0.0280), suggesting that higher lateral pre EMD/MDI were associated with a greater decrease in MDI.

### Threshold effects of medial and lateral EMD/MDI on ΔMDI

**Figure 1:**
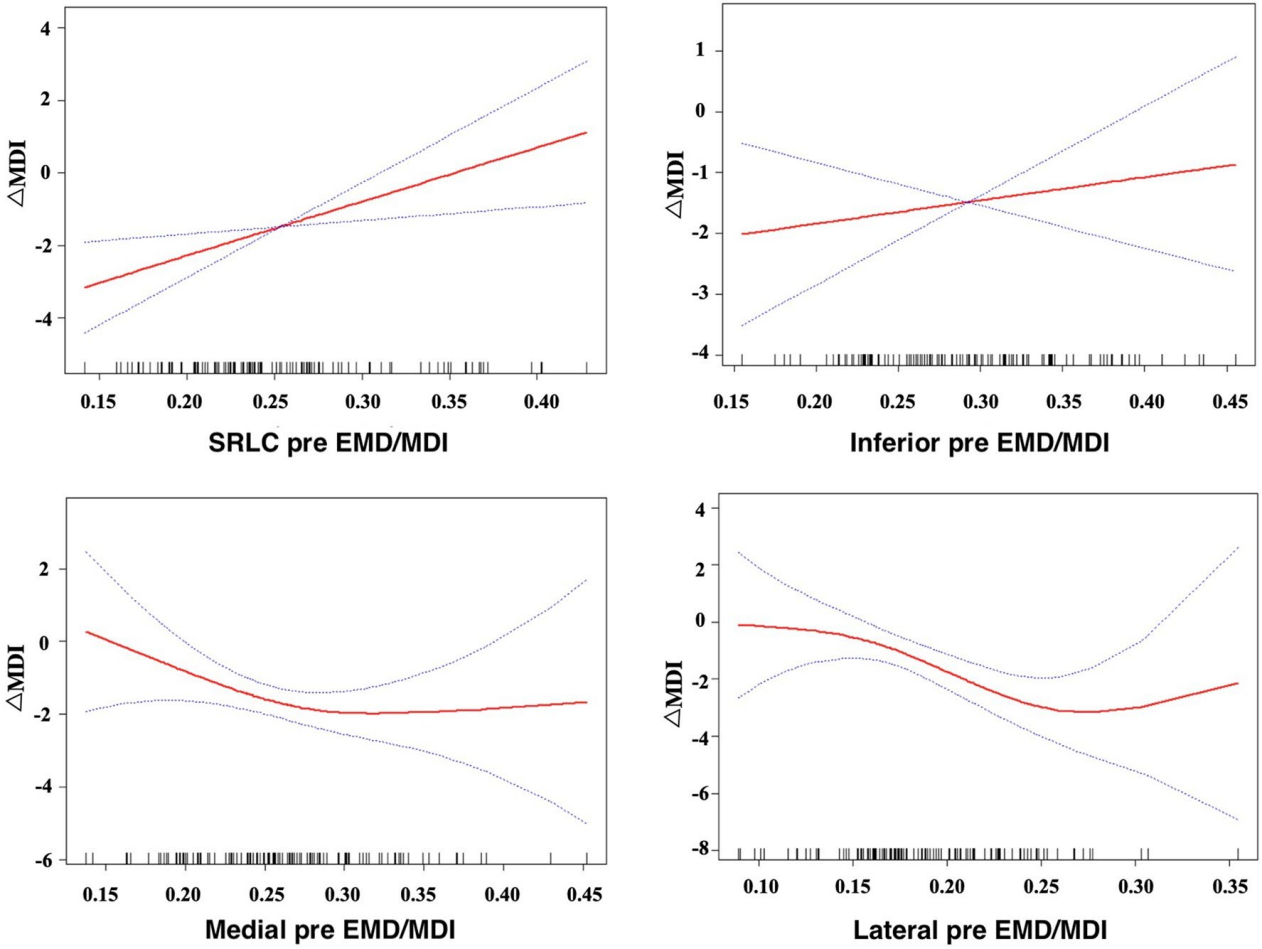
Smoothing curve fitting of pre EMD/MDI. *Notes* The solid line is the curve fitting line, and the dotted line is the 95% confidence interval.

Using smooth curve fitting and generalized summation models, we observed nonlinear relationships between medial and lateral pre EMD/MDI and ΔMDI (Fig. 1). Upon adjusting for age and gender, a threshold effect was observed between medial pre EMD/MDI, lateral pre EMD/MDI, and ΔMDI. According to the two-stage linear model and recursive algorithm, the medial pre EMD/MDI inflection point was 0.28. In the region to the left of the inflection point, a higher medial pre EMD/MDI was associated with a smaller ΔMDI (β = − 25.21, *p* = 0.0175). Conversely, no significant association between pre EMD/MDI and ΔMDI was observed in the region to the right of the inflection point. Similarly, the lateral pre EMD/MDI inflection point was identified as 0.27. In the region to the left of the inflection point, higher lateral pre EMD/MDI values were associated with a smaller ΔMDI (β = − 25.66, *p* = 0.002), while no significant correlation was observed to the right of the inflection point (Table 3).

**Table 3:** Study of the threshold effects of medial, lateral pre EMD/MDI with ΔMDI.

	Medial pre EMD/MDI	Lateral pre EMD/MDI
	β (95%CI) *P*-value	β (95%CI) *P*-value
Inflection Point(K)	0.28	0.27
< K	− 25.21 (− 45.70, − 4.71) 0.018*	− 25.66 (− 41.57, − 9.75) 0.002*
> K	13.39 (− 9.63,36.41) 0.257	52.15 (− 24.91, 129.22) 0.187

*P < 0.05; Adjusted for: age, gender.

### Association analysis between medial + lateral pre EMD/MDI and ΔMDI

The enlargement of the lateral rectus muscle alone was rare. Considering the similar trends of the association between medial and lateral pre EMD/MDI with ΔMDI and the rarity of isolated lateral rectus muscle enlargement in GO patients, we combined the medial and lateral rectus muscles to investigate their joint effect on ΔMDI. The analysis revealed a negative association between medial + lateral pre EMD/MDI and ΔMDI (β = − 11.76, *p* = 0.0188). This suggests that higher values of medial + lateral pre EMD/MDI were associated with a greater decrease in MDI values (Table 2).

## Discussion

In the present retrospective cohort study, our primary objective was to investigate the association between pre EMD/MDI and the change in MDI post-retrobulbar injection of GCs in GO patients. We analyzed data of 68 patients with 118 eyes affected by GO and administered GC injections.

Our results showed a positive correlation between SRLC pre EMD/MDI and ΔMDI. This suggests that a larger pre-existing SRLC EMD/MDI may not necessarily lead to greater differences in MDI following treatment. An explanation for this reduced response could be the potential differences in the diffusion of the drug within the SRLC compared to other muscles. This could result in a less optimal response to retrobulbar GC injections when there is significant thickening of the SRLC.

Conversely, a negative correlation was observed between the cumulative pre EMD/MDI of the medial and lateral rectus muscles and ΔMDI. This implies that patients with larger medial and lateral rectus muscle pre EMD/MDI were likely to experience larger differences in MDI following GC treatment. This could possibly be attributed to the preferential diffusion of GCs within these muscle groups, initiating a more pronounced reduction in inflammation and edema, thereby impacting the size and function of these muscles.

Interestingly, we found a threshold effect between the medial rectus pre EMD/MDI and ΔMDI. This highlights that beyond a certain point of pre-treatment MDI, no substantial additional benefits are derived from the GC therapy.

Lastly, no significant association was discovered between inferior rectus pre EMD/MDI and ΔMDI. The absence of a significant correlation might be attributed to the inherent physiological characteristics of the inferior rectus muscle, such as potential variances in fibrous composition and metabolic activities, which might affect its response to GC treatment.

Overall, these findings offer valuable insights into the efficacy of GC injections in treating GO. Future research is needed to verify these findings, investigate the precise mechanisms underlying these associations, and explore potential implications for GO treatment strategies. It is essential to emphasize that the observed associations do not imply causality and confounding factors may influence these correlations.

Based on the results of this study, MDI decreased in 84 eyes and increased in 34 eyes in GO patients who received retrobulbar injections of GCs. It is similar to the results reported by Ebner et al.^7^. Retrobulbar injection of GCs for GO resulted in positive results.

Axial CT scans were utilized to determine the horizontal diameter of the medial rectus and lateral rectus muscles at their broadest part within the muscle belly. Within the sagittal plane, measurements were conducted to ascertain the vertical diameter of both the SRLC and the inferior rectus muscle at the most expansive point of their muscle belly. Zsolt et al. found that EOM diameter was a reliable measure of EOMs volume ^12,13^. In this study, we used EMD instead of volume and MDI instead of the total volume of EOMs. The EMD/MDI was used to understand the different patterns of EOMs enlargement.

### Correlation between pre EMD and ΔMDI

Pre EMD of medial rectus, lateral rectus, SRLC, and inferior rectus were negatively correlated with ΔMDI. This suggests that the larger the diameter of each EOM before injection, the greater the shrinkage of EOMs after injection. We considered this because a larger pre EMD implies a greater potential for shrinkage of EOMs, but its guidance for clinical decision making is not meaningful. We are more concerned about the effect of different enlargement patterns on the change of EOMs, so pre EMD/MDI is the index we are more interested in.

### Caution in using retrobulbar GCs injection for patients with predominantly enlarged medial rectus musles

Threshold effects were observed in medial pre EMD/MDI changes. The inflection point for medial pre EMD/MDI was found to be 0.28. In the region to the left of the inflection point (EMD/MDI < 0.28), there was a significant negative correlation between pre EMD/MDI and ΔMDI (β = − 25.21, *p* = 0.0175). This indicates that when the ratio of medial pre EMD/MDI is below 0.28, an increase in medial pre EMD/MDI is associated with a greater shrinkage of EOM after retrobulbar injection of GCs. However, when the ratio of medial pre EMD/MDI is over 0.28, there was no significant relationship between medial pre EMD/MDI and ΔMDI.

According to Weis and Marcel Berger et al.^14,15^, GO patients with a larger medial rectus muscle have an increased risk of dysthyroid optic neuropathy (DON). Our study found that, with a medial pre EMD/MDI greater than 0.28, the shrinkage of EOMs would not gain more benefit after retrobulbar GCs injection. An excessive percentage of medial rectus muscle enlargement may result in a higher risk of DON, while retrobulbar injection of GCs may have limited benefit. Considering that injection into the orbit can temporarily cause intraorbital crowding, the use of retrobulbar GCs injections in treating GO patients with predominantly enlarged medial rectus muscles and severe medial rectus enlargements should be carefully considered.

### Patients with simultaneous enlargement of medial + lateral rectus muscles are more likely to benefit from retrobulbar GCs injection

An analysis of 2170 GO patients by Yang M concluded that 1204 eyes (31.69%) were involved in unilateral EOMs, and 2595 eyes (68.31%) in bilateral EOMs. The inferior rectus was the most commonly involved muscle, followed by the SRLC rectus^16^, medial rectus and lateral rectus, in decreasing order of frequency^17^.Yoko et al. found that in the case of unilateral and bilateral EOMs involvement, the most common site of involvement was the inferior rectus. The medial rectus appears to be enlarged more frequently in patients with bilateral EOMs involvement^18^.

In our study, we found 36 eyes with unilateral EOMs involvement. Of these, 16 eyes had inferior rectus involvement, 11 eyes had medial rectus involvement, 6 eyes had SRLC involvement, and there was no lateral rectus involvement. 82 eyes were involved in bilateral EOMs, 54 in SRLC, 74 in inferior rectus, 73 in medial rectus, and 31 in lateral rectus. This is consistent with the study mentioned above. As mentioned earlier, medial and lateral pre EMD/MDI showed similar trend of association with ΔMDI, and the enlargement of the lateral rectus muscle alone was rare, therefore a combination of the two was performed.

It has been found that changes in medial + lateral pre EMD/MDI are negatively correlated with ΔMDI(β = − 11.76, *p* = 0.0188). Suggesting that in patients who have simultaneous involvement of both the medial and lateral rectus muscles, the higher the proportion of medial + lateral pre EMD/MDI, the greater the shrinkage of EOMs following retrobulbar injection. Robert et al. found that medial and lateral rectus muscles have similar patterns of intramuscular nerve distribution^19,20^. It may be worth further investigation in the future to determine whether the similar distribution pattern of nerves in both muscles is related to the similar response of both muscles following retrobulbar injection of GCs.

### Positive correlation between SRLC enlargement and decreased EOMs volume following retrobulbar GCs injection

The results revealed a positive correlation between SRLC pre EMD/MDI and ΔMDI (β = 11.92, *p* = 0.0401). This suggests that GO patients with predominantly enlarged SRLC are more likely to experience non-shrinking EOMs after retrobulbar injection of GCs.

SRLC may also benefit from GCs injection but percentage contribution of its decrease in muscle size will be of lesser significance compared to other muscles or total MDI specially in setting of its significant enlargement. Amount of shrinkage of SRLC muscle post injection will show decreasing trend with increase in muscle size till pre SRLC/MDI is than 0.25. So, it should be emphasised that GO patients with predominantly enlarged SRLC are more likely to experience non-shrinking EOMs after retrobulbar injection of GCs.

### No significant association between inferior rectus enlargement and changes in EOMs volume after GCs injection

Additionally, we conducted linear and nonlinear correlation analyses of inferior pre EMD/MDI and ΔMDI. However, neither association showed statistical significance. It appears that the percentage of enlargement of the inferior rectus muscle does not significantly impact the changes in EOMs after retrobulbar injection of GCs. The lack of significant correlation could potentially be due to the innate physiological properties of the inferior rectus muscle, such as possible differences in its fibrous makeup and metabolic functions, which could influence how it responds to GC treatment.

### Novelty and contribution to existing literature

We underscore the originality of our study, which investigated distinct patterns of extraocular muscle (EOM) enlargement and their subsequent effects following retrobulbar GCs injection in individuals with GO. Amidst the limited presence of similar investigations within the existing literature, our research uniquely delved into the intricate interplay between specific muscle enlargement patterns and consequent changes in muscle volume. This distinctive approach augments the contemporary comprehension of outcomes associated with retrobulbar GCstreatment in GO. By offering a fresh perspective, our study enriches the scientific discourse in this domain and potentially informs clinical decision-making for patients afflicted by this condition.

### Advantages and limitations

The main advantages of this study are as follows. (1) CT measurements were used to obtain data for both orbits, ensuring the accuracy of EMD measurements. (2) In contrast to previous cross-sectional studies and case–control studies, the present study is a cohort study. It is, however, an observational study. Consequently, it is inevitably subject to confounding factors, but we rigorously adjusted for them and assessed their robustness through sensitivity analysis. The nature of observational studies limits our ability to determine causality. We can only adjust for measurable confounders, not unmeasurable ones. Hence, we should conduct larger clinical studies and at higher levels of evidence to validate our findings.

The limitations of this study are as follows: This study focused on the effects of retrobulbar injection of GCs on EMD and MDI in patients with GO. While our findings provide valuable insights into the relationship between pre EMD/MDI and changes in EOMs, it is important to acknowledge that this study did not directly compare the outcomes of retrobulbar injection with intravenous steroid treatment or radiotherapy. Therefore, the specific advantages and disadvantages of retrobulbar injection in comparison to other treatment modalities should be interpreted with caution, and further research or clinical trials comparing these treatment approaches may be warranted to fully understand their relative efficacy and limitations.

## Conclusion

In this retrospective study, we collected clinical data from GO patients who received retrobulbar GCs injections and conducted CT measurements before and after the injections. We analyzed the correlation between different patterns of EOMs enlargement and changes in EOMs volume. The results revealed an association between pre EMD/MDI and changes in EOMs following retrobulbar injection of GCs in GO patients. Specifically, in patients with predominantly enlarged medial rectus muscles and severe degrees of enlargement, retrobulbar GCs injection should be carefully evaluated for its potential benefits. Additionally, a combination of medial and lateral rectus muscle enlargement showed positive effects on EOMs shrinkage after the injections. On the other hand, SRLC enlargement is not beneficial to EOMs shrinkage following retrobulbar injections.

Our findings provide clinicians with a simple and feasible assessment method to aid in the decision-making process in clinical practice. To enhance the accuracy of this approach, future studies with larger sample sizes and broader patient populations are needed. A multicenter prospective randomized controlled clinical study with an expanded sample size would be an ideal approach to further investigate this topic.

## Data Availability

The datasets used and/or analyzed during the current study are available from the corresponding author on reasonable request.

## Abbreviations

GCs: Glucocorticoids
GO: Graves ophthalmopathy
EOM: Extraocular muscle
EMD: Extraocular muscle diameter
MDI: Diameter index
CT: Computed tomography
SRLC: Superior rectus muscle-levator complex
TA: Trimethoprim acetonide
DON: Dysthyroid optic neuropathy
